# Piezo1 Regulates ZnT1-Mediated Zinc Homeostasis in Ulcerative Colitis

**DOI:** 10.1007/s10753-025-02448-5

**Published:** 2026-01-26

**Authors:** Weizhen Xiang, Xiaoyuan Ge, Luyao Gao, Xinwen Chen, Luyao Zhang, Qiuyuan Liu, Wei Han, Qiao Mei

**Affiliations:** 1 Department of Gastroenterology, The First Affiliated Hospital of Anhui Medical University, Hefei, People’s Republic of China; 2 School of Pharmaceutical Sciences, Anhui Medical University, Hefei, People’s Republic of China

**Keywords:** Piezo1, ZnT1, Zinc homeostasis, Intestinal barrier, Ulcerative colitis

## Abstract

Ulcerative colitis (UC) is characterized by inflammatory damage of the intestinal epithelium, contributed by activation of Piezo-type mechanosensitive ion channel component 1 (Piezo1) and dysregulation of zinc homeostasis, critically maintained by zinc transporter 1 (ZnT1). Interactions between Piezo1 and ZnT1-mediated zinc homeostasis and effects on barrier function during colitis were investigated in this study. Inflammation-induced barrier disruption was modeled in vivo using dextran sulfate-treated mice and in vitro using lipopolysaccharide-treated Caco-2 cells. Intestinal epithelial cell (IEC)-specific *Piezo1* knockout in mice, small interfering RNA-mediated silencing, Piezo1 agonist and antagonist, zinc ion (Zn^2+^) supplement and chelator in Caco-2 cells were utilized to manipulate activities of Piezo1 and ZnT1. Suppression of Piezo1 in IECs alleviated inflammation-induced barrier damage in vitro and in vivo. Bioinformatic analysis of UC patient datasets revealed an association between Piezo1 and zinc homeostasis and verified by elevated Piezo1 and ZnT1 expressions observed in both human UC and murine colitis samples. Suppression of Piezo1 led to ZnT1 downregulation in vitro and in vivo*.* Piezo1 activation caused enhanced Zn^2+^ efflux and intracellular Zn^2+^ depletion while Piezo1 inhibition led to Zn^2+^ accumulation. Effects of zinc supplementation and chelation were consistent with the view that ZnT1 inhibition alleviated barrier disintegration. Our findings revealed that Piezo1 affected ZnT1-mediated zinc homeostasis in IECs. Piezo1 suppression downregulated ZnT1 expression, attenuating intracellular zinc depletion caused by excessive Zn^2+^ efflux and relieved intestinal barrier disruption, indicating that Targeting the Piezo1-ZnT1-zinc signaling axis presents a promising therapeutic strategy for UC.

**Graphical Abstract:**

Piezo1-activation exacerbates inflammation-induced intestinal barrier disruption by triggering Ca^2+^ influx which upregulates ZnT1-mediated Zn^2+^ efflux resulting in IECs Zn^2+^ dyshomeostasis, and this disruption can be rescued by Piezo1 suppression. The figure was generated by adobe illustrator.

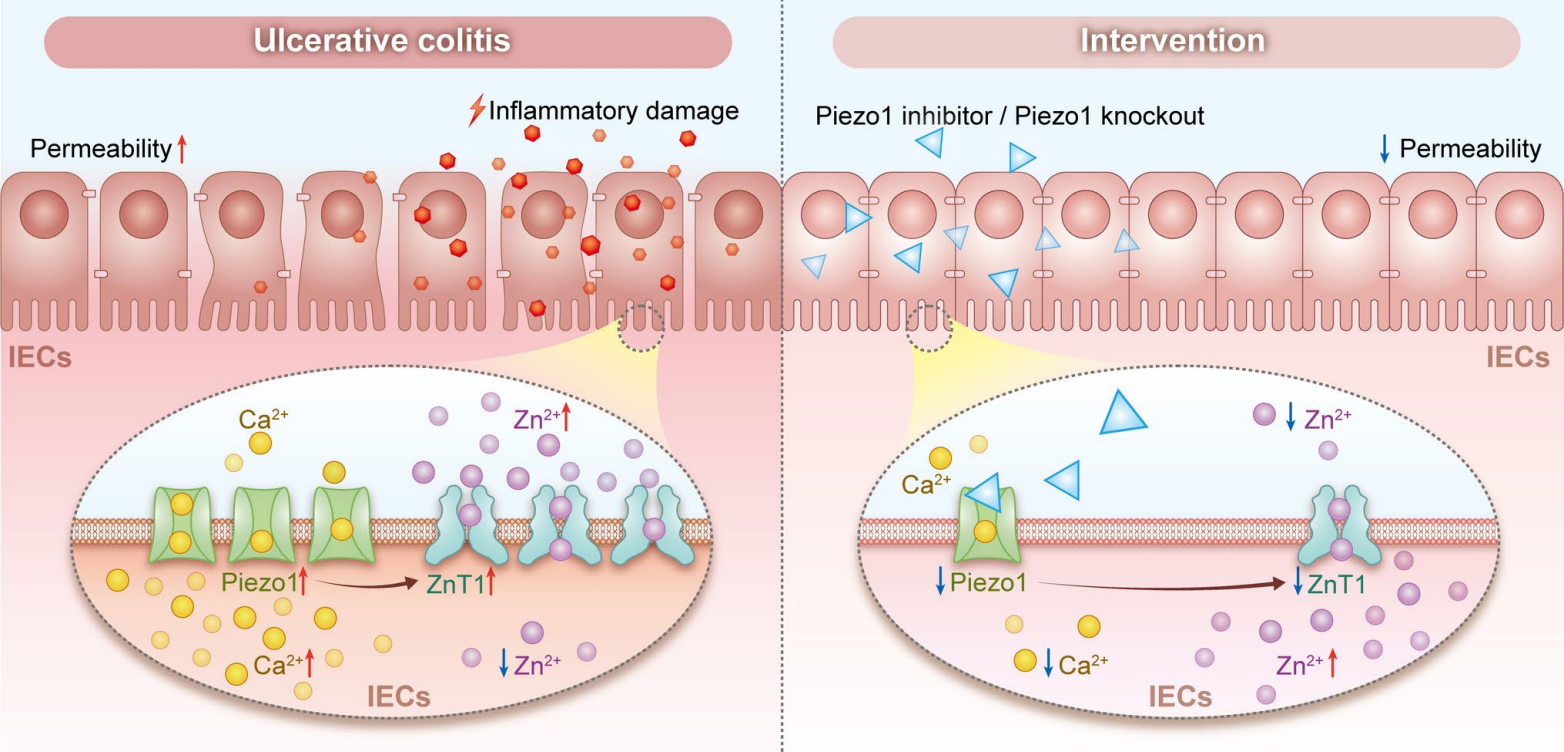

**Supplementary Information:**

The online version contains supplementary material available at 10.1007/s10753-025-02448-5.

## Introduction

Ulcerative colitis (UC) is a chronic inflammatory bowel disease (IBD), characterized by recurrent colonic and rectal mucosal inflammation which leads intestinal barrier disruption [1]. UC imposes a heavy clinical burden, since around 5 million patients are affected worldwide and existing therapies are limited, with remission rates of 30%−60% and the requirement for surgery by the 10%−20% of refractory cases [1, 2]. The underlying pathological mechanisms of UC remain poorly understood, requiring further exploration in order to improve the prognosis of this disease.

Recent advances indicate a role for Piezo-type mechanosensitive ion channel component 1 (Piezo1) in intestinal homeostasis [3]. This protein is expressed in intestinal epithelial cells (IECs) and converts the mechanical stimuli of pressure, shear stress or stretch into downstream ion signals, participating in immunity, cell proliferation and barrier function [3]. The upregulation of Piezo1 has been reported in the intestinal mucosa of IBD patients [4–6] and inhibition shown to ameliorate inflammatory damage in experimental colitis models [4, 7, 8].

Transcriptomic analysis of IEC-specific Piezo1 knockout (*Piezo1*^ΔIEC^) and wild type (WT) mice showed altered mineral absorption and intracellular zinc homeostasis (unpublished data), suggesting an impact of Piezo1 on zinc balance in IECs. Zinc homeostasis is known to be involved in the maintenance of intestinal barrier integrity. IBD patients often suffered from zinc deficiency and supplementation alleviated mucosal inflammation in both patients and animal models [9–11]. Zinc ions (Zn^2+^) have been reported to modulate epithelial barrier function and expression of tight junction (TJ) proteins in IECs [12–15]. Intracellular zinc homeostasis is mainly maintained by the SCL30A/Zn (ZnT) family of transporters which mediate efflux and SLC39A/Zrt- and Irt-like proteins (ZIP) which mediate influx [16]. Among these, Zinc transporter 1 (ZnT1) is a crucial zinc transporter located on IEC plasma membrane responsible for export Zn^2+^ to extracellular space, and its expression level would change to.

prevent cytotoxic Zn^2+^ overload or deficiency [16]. The homeostatic role of ZnT1 raises the possibility that aberrant ZnT1 overexpression in UC depletes cytoprotective zinc pools, resulting in barrier damage.

The current study investigated the role of abnormal Piezo1 activation in stimulating ZnT1-mediated zinc homeostasis in IECs during inflammation, aiming to elucidate a novel Piezo1-ZnT1-zinc signaling axis contributing to UC pathogenesis with therapeutic potential.

## Materials and Methods

### Human Samples

Human samples were sourced from the Department of Gastroenterology of the First Affiliated Hospital of Anhui Medical University, colonic mucosal biopsy specimens from 6 UC patients and 6 healthy controls were collected during colonoscopic examination. Experiments involving patient samples and data were conducted in accordance with the Declaration of Helsinki. Written informed consent was given by all participants for the use of specimens and publication of data and ethical approval was granted by the ethics committee of Anhui Medical University (approval no. PJ 2025–02–51). All patients were newly diagnosed without prior treatment, the UC diagnosis was established through comprehensive assessment, including clinical features, endoscopic and radiological findings, histopathological examination, and exclusion of alternative diagnoses [17]. Basline characteristics of patients and comntrols are available in Table S1.

### Animal Model

Conditional Piezo1 knockout mice (*Piezo1*^flox/flox^, C57BL/6Smoc-Vil1em(CreERT2)2Smoc, Cat. No. NM-CKO-200275) and intestinal epithelial cell-specific inducible Cre mice (*Vil*^CreERT2/+^, C57BL/6Smoc-Vil1em(CreERT2)2Smoc, Cat. No. NM-KI-225020) on C57BL/6 background were purchased from Shanghai Model Organisms Center (Shanghai, China) [6, 18]. IEC-specific Piezo1 knockout mice (*Piezo1*^ΔIEC^, *Piezo1*^flox/flox^; *Vil*^CreERT2/+^, see Fig. S1a-d for knockout validation) were generated by crossing these two strains, and the *Piezo1* deletion was induced in 6–8-week-old mice by intraperitoneal injection of 120 mg/kg tamoxifen (Cat. No. ST1681, Beyotime, Shanghai, China) for five times, once every other day [19]. Littermate *Piezo1*^flox/flox^ mice (WT) were used as controls. Animals were housed in specific pathogen-free facilities at Laboratory Animal Center, Anhui Medical University with autoclaved food and water ad libitum. Male WT and *Piezo1*^ΔIEC^ mice, aged 6–8 weeks, were randomly assigned to four groups (*n* = 6): WT (no dextran sulfate sodium (DSS) treatment), *Piezo1*^ΔIEC^ (no DSS treatment), WT-DSS (DSS treatment) and *Piezo1*^ΔIEC^-DSS (DSS treatment). DSS (Cat. No. 160110, MP Biomedicals, Santa Ana, CA, USA) was given as a 3% (w/v) solution in drinking water for 7 consecutive days to establish the model of acute colitis [20]. Weight loss, stool consistency and fecal bleeding were assessed daily, evaluated by Disease Activity Index (DAI) scoring according to standardized criteria described by Murthy et al., 1992 [18] to confirm colitis (see Table S2). Mice were sacrificed on the eighth day and colon tissues were collected for follow-up experiments. Animal experiments were conducted in accordance with the Guidelines for the Care and Treatment of Laboratory Animals and ethical approval was granted by the ethics committee of Anhui Medical University (Approval no.20221095).

### Cell Culture and Treatment

The human rectal adenocarcinoma 2 cell line (Caco-2) was supplied by the National Collection of Authenticated Cell Cultures of the Chinese Academy of Sciences, cultured in Dulbecco's Modified Eagle Medium (Cat. No. BL301A, Biosharp, Shanghai, China) supplemented with 20% fetal bovine serum (Cat. No. BL201A, Biosharp) and 1% penicillin–streptomycin solution (Cat. No. BL301A, Biosharp) at 37℃ in 5% CO2 with medium change every 48 h. Caco-2 cells were seeded onto polycarbonate Transwell inserts (12 mm diameter, 0.336 cm2 surface area, 3.0 µm pore size; Corning, NY, USA) at a density of 4.0 × 10^5^ cells per insert, allowed to differentiate for 21 days until forming confluent monolayers. Suppression of Piezo1 and ZnT1 expression was achieved by transfection of small interfering RNAs (siRNAs) using Lipofectamine 3000 (Cat. No. L3000015, Thermo Fisher Scientific, Waltham, MA, USA). siRNAs and negative controls (si-NC) were purchased from RiboBio (Guangzhou, Guangdong, China), sequences of si-*Piezo1* and si-*ZnT1* (see Table S3) and knockdown validation (see Fig S1e,f and Fig. S2a,b) are available in supplementary materials. Caco-2 cell monolayers were stimulated with 10 μg/mL lipopolysaccharide (LPS, Cat. No. L2630, Sigma-Aldrich, St Louis, MO, USA) for 24 h to establish an inflammatory model. Caco-2 cell monolayers were treated with 100 μM ZnCl_2_ (Cat. No. 229997, Sigma-Aldrich, St Louis, MO, USA) or 10 μM zinc chelator N,N,N',N'-Tetrakis (2-pyridylmethyl)−1,2-ethylenediamine (TPEN, Cat. No. P4413, Sigma-Aldrich, St Louis, MO, USA) for 12 h prior to adding LPS stimulation or control to produce conditions of Zn^2+^ sufficiency or deficiency. All experiments were conducted in triplicate. To isolate mouse colonic epithelial cells, briefly euthanize the mouse, harvest and open the colon longitudinally, then wash and fragment the tissue. Incubate the fragments in a cold PBS/EDTA solution with shaking to slough off epithelial cells, then pellet the cells by centrifugation [21].

### Transepithelial Electrical Resistance (TEER) Measurement

After cultured for 21 days, the integrity of Caco-2 cell monolayers was validated by a steady-state TEER value of ≥ 500 Ω·cm^2^. TEER was measured by epithelial volt-ohmmeter (EVOM2, World Precision Instruments, Sarasota, FL, USA) equipped with STX-2 chopstick electrodes. Monolayers were washed twice with pre-warmed Hank’s balanced salt solution (HBSS; Cat. No. BL562A, Biosharp) before measurement and the EVOM2 calibrated, according to manufacturer guidelines. TEER (Ω·cm^2^) = (raw resistance value (Ω)—blank resistance value (Ω)) × membrane surface area (cm^2^), where blank value was resistance of cell-free inserts filled with HBSS and membrane surface area was Transwell surface area (0.336 cm^2^) [22].

### Histology

Colon biopsy samples were fixed in 4% paraformaldehyde for 24 h, dehydrated in an ethanol gradient, cleared in xylene and embedded in paraffin blocks which were sliced into 3 μm sections, mounted on glass slides and dried at 85℃ for 1 h. Inflammatory damage was evaluated by hematoxylin and eosin (H&E) staining. Sections were deparaffinized in xylene, rehydrated by ethanol gradient, nuclei stained with hematoxylin for 3 min, rinsed in running water for 5 min and cytoplasmic structures counterstained with eosin for 1 min, cleared in xylene and covered with neutral resin for imaging (Motic Easyscan, Motic, Xiamen, Fujian, China). H&E staining results were scored for inflammation severity, inflammation extent, crypt damage and percentage involvement, according to a previously published method giving a total histological score between 0–14 (see Table S4 for scoring criteria) [23].

### Immunofluorescence (IF)

3 μm sections of human and mouse colon tissue were heated to 85℃, deparaffinized with xylene and rehydrated by ethanol gradient. Samples were heated in citrate buffer in a pressure cooker for 20 min and cooled to room temperature for antigen retrieval. Incubation with 3% hydrogen peroxide and 1 M sodium hydroxide for 2 h was performed to quench autofluorescence and with 10% goat serum and at 37℃ for 1 h to block non-specific binding. Sections were incubated overnight at 4℃ with primary antibodies raised against Piezo1 (1:100;Cat. No. BL562A, Thermo Fisher Scientific), ZnT1 (1:200; Cat. No. PA5-104383, Thermo Fisher Scientific), zonula occludens-1 (ZO-1) (1:100; Cat. No. 33–9100, Invitrogen, Carlsbad, CA, USA), occludin (1:100; Cat. No. ab216327, Abcam), Alexa Fluor 647 anti-sodium potassium ATPase (NaKATPase, 1:100; Cat. No. ab198367, Abcam), followed by washing with PBS and incubation with secondary antibodies, 488-goat anti-rabbit recombinant secondary antibody (1:200; Cat. No. RGAR002, Proteintech, Rosemont, IL, USA) and 594-goat anti-mouse recombinant secondary antibody (1:200; Cat. No. RGAM004, Proteintech) at 37℃ for 45 min. Nuclei were stained and slides mounted in antifade mounting medium with DAPI (Cat. No. P0131, Beyotime, Shanghai, China) and imaged by confocal microscope (Sunny technology, Beijing, China). Fluorescence was quantified with ImageJ software (v2.0.0; National Institutes of Health, Bethesda, MD, USA). The quantitative analysis of target proteins were performed using Image J software. Each group included six samples, and for each tissue sample, five non-overlapping fields of view were randomly selected. Subsequently, the integrated density value of the fluorescence signal of each field was measured, and the expression level of the target protein was measured using the mean of these five values.

### Western Blotting (WB)

Colon tissue and Caco-2 cells were lysed in radioimmunoprecipitation assay buffer (Cat. No. P0013C, Beyotime) containing protease and phosphatase inhibitors (Cat. No. P1045, Beyotime), and protein concentration measured in the supernatant by BCA assay kit (Cat. No. P0012, Beyotime). Equal quantities of protein in loading buffer (Cat. No. P0015, Beyotime) were separated by sodium dodecyl sulfate–polyacrylamide gel electrophoresis and electro-transferred onto PVDF membranes (Millipore, Billerica, MA, USA). Membranes were blocked with 5% skimmed milk and incubated overnight at 4℃ with primary antibodies raised against Piezo1 (1:2000; Thermo Fisher Scientific), ZnT1 (1:2000; Thermo Fisher Scientific), ZO-1 (1:2000; Invitrogen), occludin (1:2000; Abcam), glyceraldehyde-3-phosphate dehydrogenase (GAPDH) (1:50000; Cat. No. 60004–1-Ig, Proteintech), washed with tris-buffered saline with tween-20 and incubated with secondary antibodies, HRP-conjugated goat anti-mouse IgG (1:5000; Cat. No. SA00001-1, Proteintech) and HRP-conjugated goat anti-rabbit IgG (1:5000; Cat. No. SA00001-2, Proteintech) for 1 h at room temperature. Protein bands were visualized with enhanced chemiluminescence substrate (Tanon, Shanghai, China) on a Tanon-5200 imaging system and quantified using ImageJ software (v2.0.0).

### Quantitative Real-Time Polymerase Chain Reaction (qRT-PCR)

Total RNA was isolated from colon tissue and cultured cells using TRIzol purification kit (Cat. No. 12183555, Invitrogen) and concentration and purity determined by Nanodrop Spectrophotometer (Thermo Fisher Scientific). Complementary DNA was synthesized from total RNA using HiScript III RT SuperMix for qPCR (Cat. No. R323, Vazyme, Nanjing, Jiangsu, China), qRT-PCR performed in ChamQ SYBR qPCR Master Mix (Cat. No. Q421, Vazyme) on an Applied Biosystems StepOne Real-Time PCR System (Applied Biosystems, Carlsbad, CA, USA). RNA expression was normalized to GAPDH and relative expression calculated using the 2^−ΔΔCT^ method. See Table S5 for primer sequences.

### Zinc Imaging and Calcium Imaging

Incubated Caco-2 monolayers with 1 µM FluoZin-3 (Cat. No. F24195, Invitrogen, Carlsbad, CA, USA) or mice colonic epithelial cells with 5 µM Fluo-4AM (Cat. No. S1060, Beyotime) for 30 min at 37℃, washed three times in fresh HBSS and fluorescence measured by Olympus inverted fluorescence microscope. Changes in sample fluorescence intensity were considered to indicate changes in Zn^2+^ or calcium ion (Ca^2+^) concentration [24, 25]. 5 μM Piezo1 channel activator, Yoda1 (Cat. No. 5586, Bio-Techne, Minneapolis, MN, USA), or 5 μM channel inhibitor, Grammostola spatulata mechanotoxin 4 (GsMTx4, Cat. No. ab141871, Abcam), were added after stabilization of baseline fluorescence value. 3 mM Ca^2+^ supplement, calcium chloride (CaCl_2_, Cat. No. Y001521, Beyotime) or 3 mM Ca^2+^ chelator, ethylenediaminetetraacetic acid (EDTA, Cat. No. ST1303, Beyotime), were incubated with Caco-2 monolayers for 30 min before Yoda1 treatment if necessary.

### Bioinformatic Analysis of Gene Expression Omnibus (GEO) Datasets

The microarray dataset, *GSE73661*, of paired samples from 166 UC patients and 12 healthy controls was downloaded from GEO (https://www.ncbi.nlm.nih.gov/geo/). Data processing, statistical analysis and visualization were performed in R programming (v4.5.0; Posit PBC, Boston, MA, USA) and language packages used were, “GEOquery” (v2.70.0) for data downloading, “AnnoProbe” (v0.2.8) for probe annotation, “dplyr” (v1.1.4) for data cleaning and “preprocessCore” (v1.64.0) for data normalization. Box plot showing different Piezo1 expression between UC and controls was generated by “ggpubr” package (v0.6.0). “limma” package (v3.58.1) was used for analysis of differentially expressed genes (DEGs) between low and high Piezo1 expressing UC patients. DEGs were defined as having an absolute logarithmic value of the fold change (log_2_FC) in expression level between two groups > 1 and adjusted p-value < 0.05. Volcano plot and heat map illustrating DEG results were generated by “ggplot2” package (v3.5.0) and “pheatmap” (v1.0.12), respectively. “clusterProfiler” (v4.10.3) was used for Gene Ontology (GO) and Kyoto Encyclopedia of Genes and Genomes (KEGG) enrichment analysis of DEGs and results visualized by bubble plot via “ggplot2”. Significantly enriched pathways were defined as -log10(*p*-value) > 1.3.

### Statistical Analysis

Statistical analyses were performed using GraphPad Prism (v10.5.0; GraphPad Software, San Diego, CA, USA) software. Quantitative data resulting from at least 3 independent experiments are presented as mean ± standard deviation (SD) for normally distributed variables or median (interquartile range, IQR) for non-normally distributed variables. Inter-group comparisons of normally distributed data were made by two-tailed unpaired t-test for two groups and by one-way analysis of variance (ANOVA) followed by Student–Newman–Keuls post hoc test for multi-group comparisons. Non-normally distributed data were analyzed non-parametrically by Mann–Whitney U test for two groups and Kruskal–Wallis with Dunn’s test for multiple groups. Statistical significance was defined as *p* < 0.05 for all comparisons. Graphical representations were generated by GraphPad Prism.

## Results

### Piezo1 Suppression Alleviated Inflammation-Induced Colonic Epithelial Barrier Disruption

**Fig. 1:**
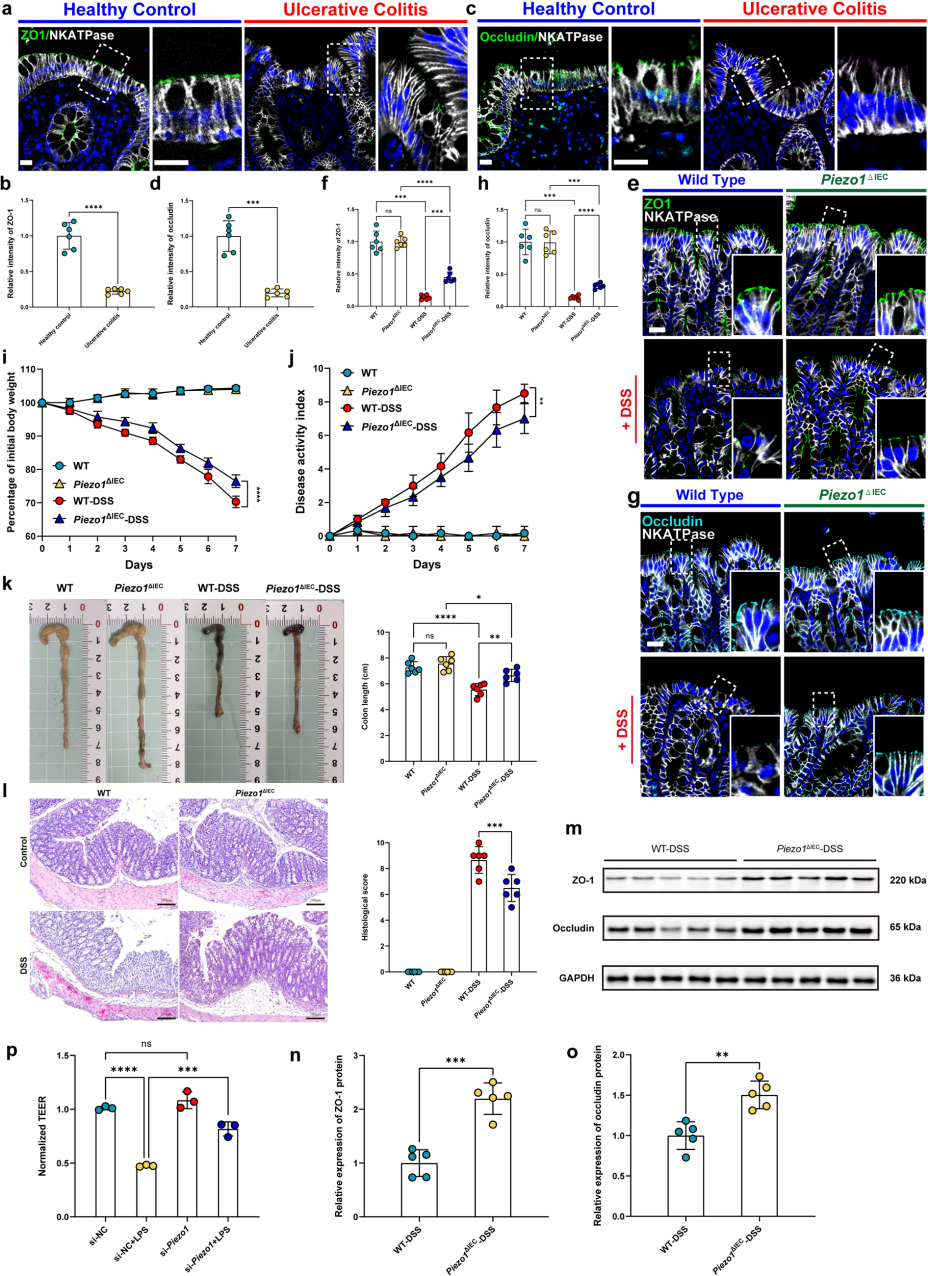
Piezo1 suppression alleviated inflammation-induced colonic epithelial barrier disruption. **a** Representative images showing ZO-1 IF staining of human colon tissues in healthy controls and UC patients. Scale bar: 20 μm. **b** Quantitative analysis of images shown in (a); *n* = 6 each group. **c** Representative images showing occludin IF staining of human colon tissues in healthy controls and UC patients. Scale bar: 20 μm. **d** Quantitative analysis of images shown in (c); *n* = 6 each group. **e** Representative images showing ZO-1 IF staining of mouse colon tissues from groups WT, Piezo1^ΔIEC^, WT-DSS and *Piezo1*^ΔIEC^ -DSS. Scale bars: 20 μm. **f** Quantitative analysis of images shown in (e); *n* = 6 each group. **g** Representative images showing ZO-1 IF staining of mouse colon tissues from groups WT, *Piezo1*^ΔIEC^, WT-DSS and *Piezo1*^ΔIEC^-DSS. Scale bars: 20 μm. **h** Quantitative analysis of images shown in (g); *n* = 6 each group. **i** Time course of body weight changes of mice from groups WT, *Piezo1*^ΔIEC^, WT-DSS and *Piezo1*^ΔIEC^-DSS over 7 days of treatment; *n* = 6 each group. **j** Time course of DAI changes of mice from groups WT, *Piezo1*^ΔIEC^, WT-DSS and *Piezo1*^ΔIEC^-DSS over 7 days of treatment; *n* = 6 each group. **k** Representative images and colon length statistics of mice from groups WT, *Piezo1*^ΔIEC^, WT-DSS and *Piezo1*^ΔIEC^-DSS sacrificed at day 8; *n* = 6 each group. **l** Representative images H&E staining and histological scoring statistics of mouse colon tissues from groups WT, *Piezo1*^ΔIEC^, WT-DSS and *Piezo1*^ΔIEC^-DSS sacrificed at day 8; *n* = 6 each group. **m** Representative images of WB showing ZO-1 and occludin protein expression in mouse colon tissues from groups WT, *Piezo1*^ΔIEC^, WT-DSS and *Piezo1*.^ΔIEC^-DSS; *n* = 5 each group. **n** and **o** Quantitative analysis of images shown in (m); *n* = 5 each group. **p** TEER values of Caco-2 cells transfected with si-*Piezo1* or si-NC and stimulated with LPS or not; *n* = 3 each group. Data are presented as mean ± SD (error bars). *p < 0.05, **p < 0.01, ***p < 0.001 and ****p < 0.0001

The expression of TJ proteins, ZO-1 and occludin, was markedly reduced in colonic mucosa of UC patients and DSS-treated mice relative to healthy or untreated controls, demonstrated by IF staining (Fig. 1a-h). *Piezo1* deletion in IECs in DSS-induced murine colitis resulted in lower weight loss, lower DAI scores, reduced colon shortening and milder histological damage (Fig. 1i-l), indicating a reduced degree of disease severity. Additionally, after withdrawing DSS treatment, the recover rate of body weight of *Piezo1*^ΔIEC^-DSS mice was significantly higher than WT-DSS mice during a 7-day recovery period after DSS withdrawal (Fig S3a), the colon length of *Piezo1*^ΔIEC^-DSS group were also significant longer than WT-DSS group on the 7th day after the withdrawal of DSS treatment (Fig S3b). Comparison of WT-DSS with *Piezo1*^ΔIEC^-DSS group showed higher expression of ZO-1 and occludin in colonic tissue after Piezo1 deletion assessed by IF and WB analysis, indicating its protective effect on intestinal barrier function (Fig. 1m-o). *Piezo1* knockdown by si-*Piezo1* RNA transfection of Caco-2 cells showed attenuated TEER reduction resulting from LPS treatment compared with si-NC transfected cells (Figs. 1p), supporting the in vivo findings that *Piezo1* suppression alleviated inflammation-induced epithelial barrier damage.

### Piezo1 Expression was Associated With Zinc Homeostasis in UC

**Fig. 2:**
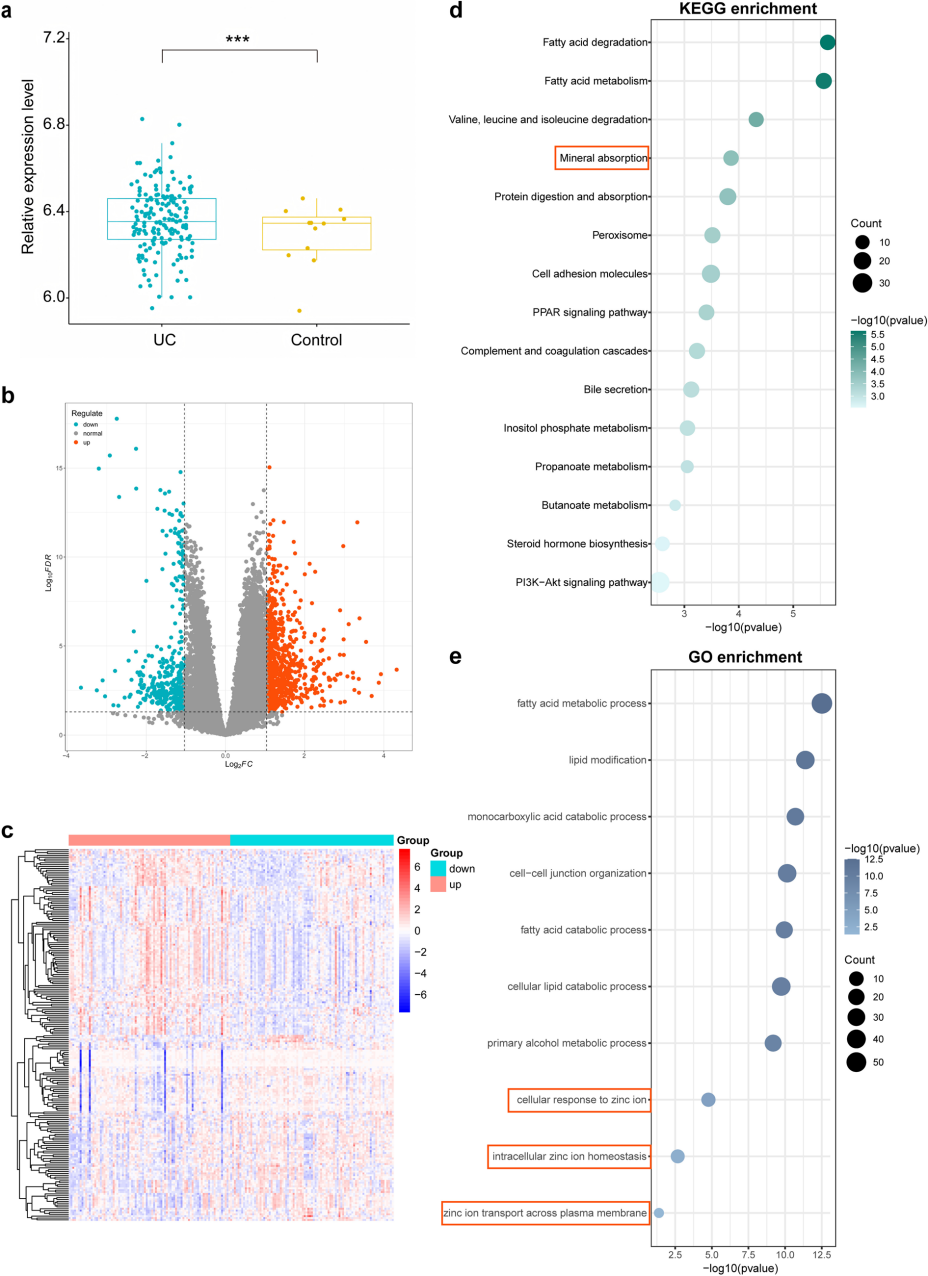
Piezo1 expression was associated with zinc homeostasis in UC. **a** Box plot showing Piezo1 expression in colon biopsies from UC patients (*n* = 166) and healthy controls (*n* = 12) from the *GSE73661* dataset (***p < 0.001). **b** Volcano plot showing DEGs between UC samples with low and high Piezo1 expression divided according to a threshold of median Piezo1 expression, refer to Table S6 for the gene symbols of those genes shown in the figure. **c** Heat map showing top 50 up/down-regulated DEGs in high- and low-expressing UC samples, refer to Table S6 for the gene symbols of those genes shown in the figure. **d** KEGG enrichment analysis of DEGs, refer to Table S7 for the detailed information of highlighted pathways. **e** GO enrichment analysis of DEGs, refer to Table S8 for the detailed information of highlighted pathways. Graduated green/blue color bar indicates significance of differential expression with a higher intensity color indicating greater significance. Dot size represents numbers of DEGs per pathway with a larger dot indicating greater gene involvement

Analysis of dataset *GSE73661* showed higher *Piezo1* expression in colon biopsies from UC patients compared with healthy controls (Fig. 2a). Stratification of UC patients into high- and low-*Piezo1* expression based on a threshold of median expression showed that 658 genes were differentially expressed between the two groupings, 256 downregulated and 402 upregulated. Differentially expressed genes (DEGs) are represented as a volcano plot (Fig. 2b) and the top 50 DEGs as a heatmap (Fig. 2c). KEGG analysis revealed DEGs to be enriched in pathways linked to mineral absorption (Fig. 2d) and GO analysis showed enrichment in pathways related to cellular response, intracellular Zn^2+^ homeostasis and Zn^2+^ transport across the plasma membrane (Fig. 2e). The data above demonstrates that elevated *Piezo1* expression in UC samples is likely to be associated with intracellular zinc dyshomeostasis.

### Zinc Homeostasis and ZnT1 Suppression Preserved Epithelial Barrier Integrity During Inflammation

**Fig. 3:**
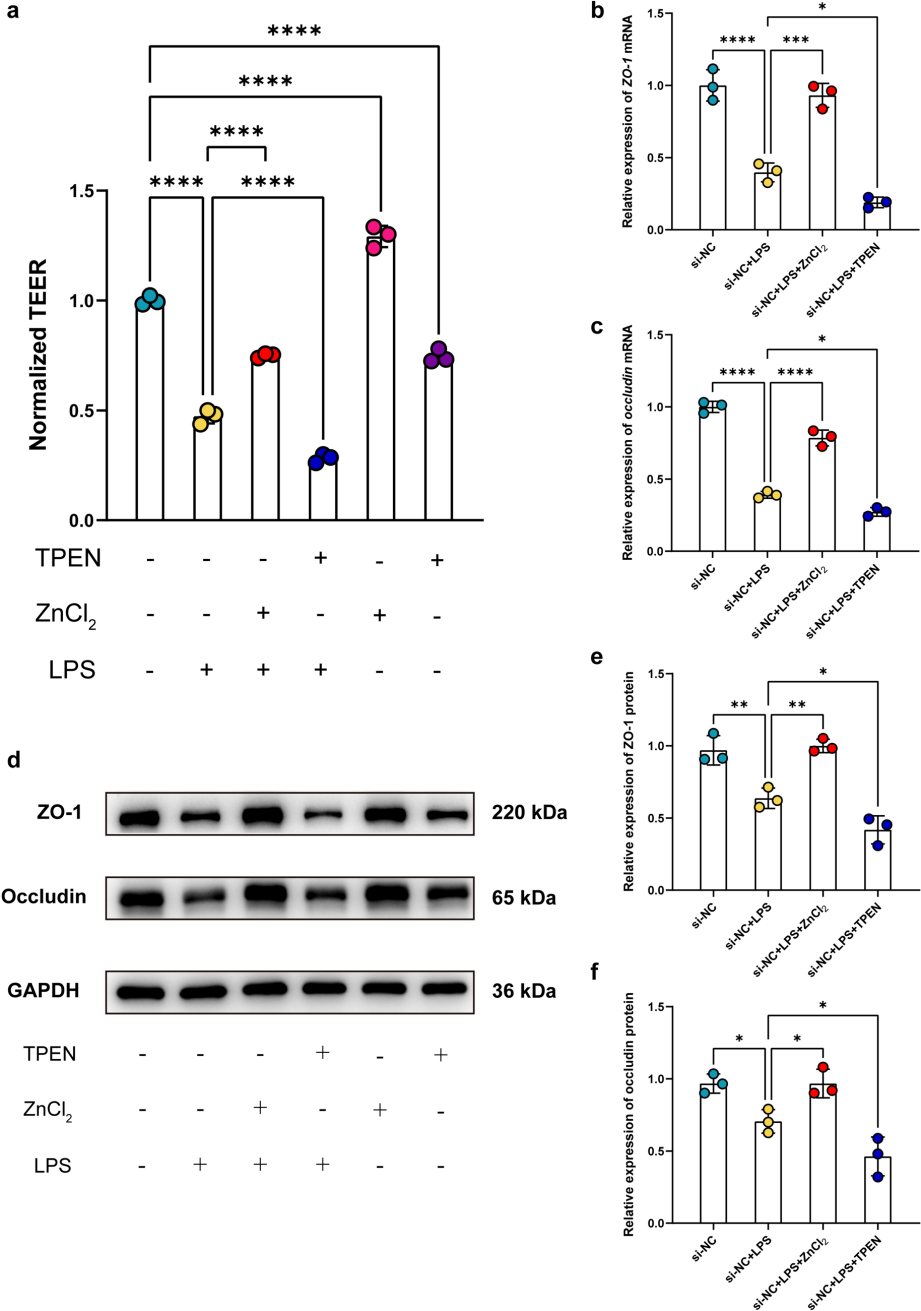
Zinc homeostasis preserved epithelial barrier integrity during inflammation. **a** TEER values of Caco-2 cells stimulated with LPS or not and treated with ZnCl_2_ and/or TPEN or not; *n* = 3 each group. **b** qRT-PCR analysis of *ZO-1* mRNA expression in Caco-2 cells stimulated with LPS or not and treated with ZnCl_2_ or TPEN or not; *n* = 3 each group. **c** qRT-PCR analysis of *occludin* mRNA expression in Caco-2 cells stimulated with LPS or not and treated with ZnCl_2_ or TPEN or not; *n* = 3 each group. **d** Representative images of WB showing ZO-1 and occludin expression in Caco-2 cells stimulated with LPS or not and treated with ZnCl_2_ and/or TPEN or not. (**e**) Quantitative analysis of ZO-1 protein expression in Caco-2 cells stimulated with LPS or not and treated with ZnCl_2_ or TPEN or not; *n* = 3 each group. (f) Quantitative analysis of occludin protein expression in Caco-2 cells stimulated with LPS or not and treated with ZnCl_2_ or TPEN or not; *n* = 3 each group. Data are presented as mean ± SD (error bars). *p < 0.05, **p < 0.01, ***p < 0.001 and ****p < 0.0001

**Fig. 4:**
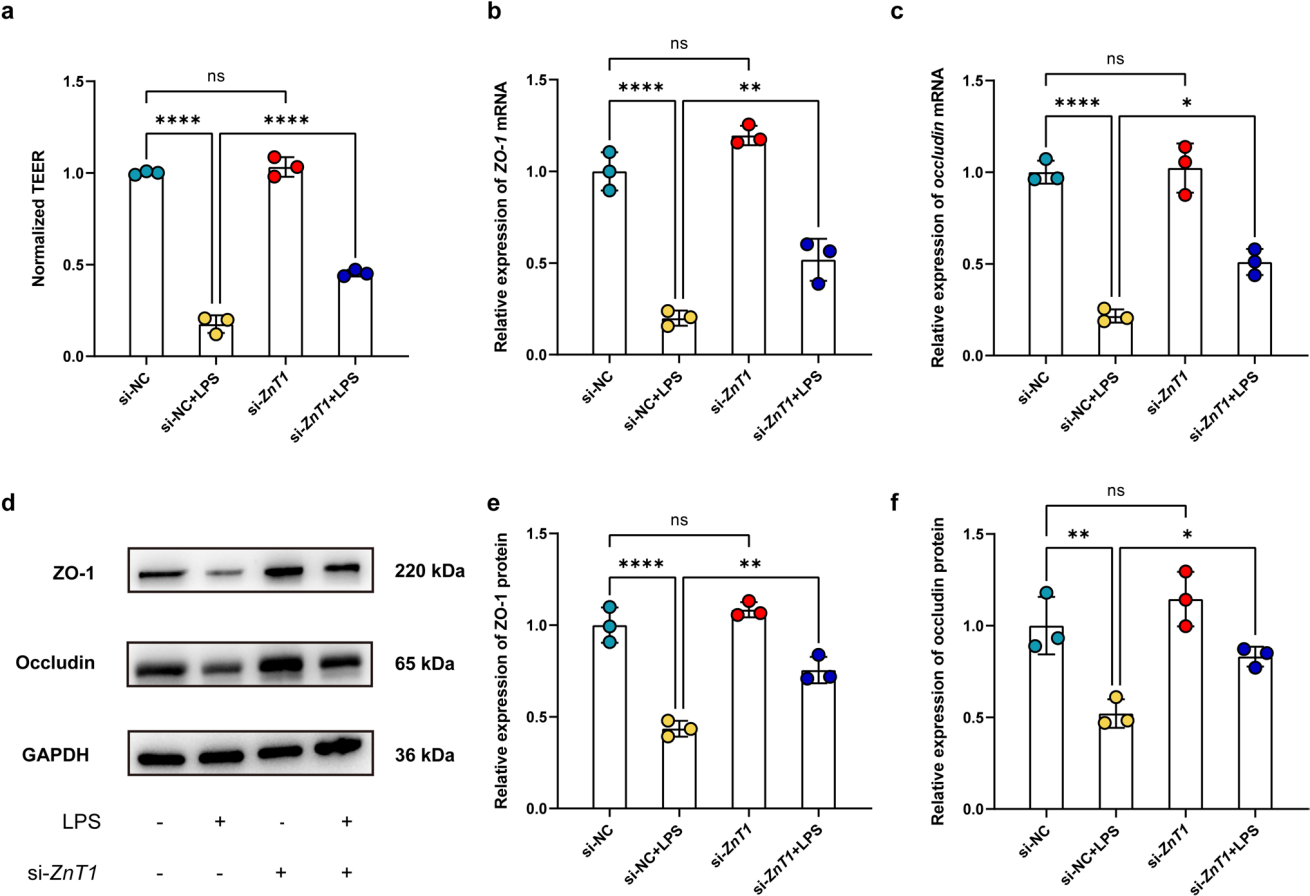
ZnT1 suppression attenuated inflammation-induced epithelial barrier disruption. **a** TEER values of Caco-2 cells transfected with si-*ZnT1* or si-NC and stimulated with LPS or not; *n* = 3 each group. **b** qRT-PCR analysis of *ZO-1* mRNA expression in Caco-2 cells transfected with si-*ZnT1* or si-NC and stimulated with LPS or not; *n* = 3 each group. **c** qRT-PCR analysis of occludin mRNA expression in Caco-2 cells transfected with si-*ZnT1* or si-NC and stimulated with LPS or not; *n* = 3 each group. **d** Representative images of WB showing ZO-1 and occludin expression in Caco-2 cells transfected with si-*ZnT1* or si-NC and stimulated with LPS or not. **e** Quantitative analysis of ZO-1 protein expression in Caco-2 cells transfected with si-*ZnT1* or si-NC and stimulated with LPS or not; *n* = 3 each group. **f** Quantitative analysis of occludin protein expression in Caco-2 cells transfected with si-*ZnT1* or si-NC and stimulated with LPS or not; *n* = 3 each group. Data are presented as mean ± SD (error bars). *p < 0.05, **p < 0.01, ***p < 0.001 and ****p < 0.0001

Zinc supplementation via pretreatment with exogenous Zn^2+^ to Caco-2 monolayers (si-NC + LPS + ZnCl_2_) attenuated LPS-induced TEER reduction compared with the control group (si-NC + LPS), whereas zinc depletion caused by the Zn^2+^ chelator TPEN (si-NC + LPS + TPEN) exacerbated TEER reduction (Fig. 3a), indicating that Zn^2+^ has a protective effect on the maintenance of barrier integrity under conditions of inflammation. WB and qRT-PCR also showed that zinc supplementation (si-NC + LPS + ZnCl_2_) alleviated the reduction of mRNA and protein expression of TJ proteins, ZO-1 and occludin, caused by LPS stimulation (Fig. 3b-f). On the contrary, zinc chelation (si-NC + LPS + TPEN) aggravated the reduction of ZO-1 and occludin expression (Fig. 3b-f). Thus, it can be concluded that the maintenance of epithelial barrier integrity under inflammatory conditions relies on appropriate intracellular zinc homeostasis. To further explore the regulatory mechanism of zinc in barrier function, we knocked down *ZnT1* via siRNA transfection. *ZnT1* knockdown in Caco-2 cells (si-*ZnT1* + LPS) ameliorated the decreased TEER resulting from LPS stimulation (si-NC + LPS) (Fig. 4a). WB and qRT-PCR analysis revealed consistent results that protein and mRNA expression levels of TJ proteins, ZO-1 and occludin, were significantly higher in si-ZnT1 group (si-*ZnT1* + LPS) compared with controls after LPS treatment (si-NC + LPS) (Fig. 4b-f). The attenuating effect of ZnT1 suppression was consistent with zinc supplementation on inflammation-induced epithelial barrier disruption, suggesting that ZnT1 activation during inflammatory conditions may contribute to impaired barrier function by dysregulating zinc homeostasis.

### ZnT1 Expression was Elevated in Colitis and Correlated With Piezo1 Expression

**Fig. 5:**
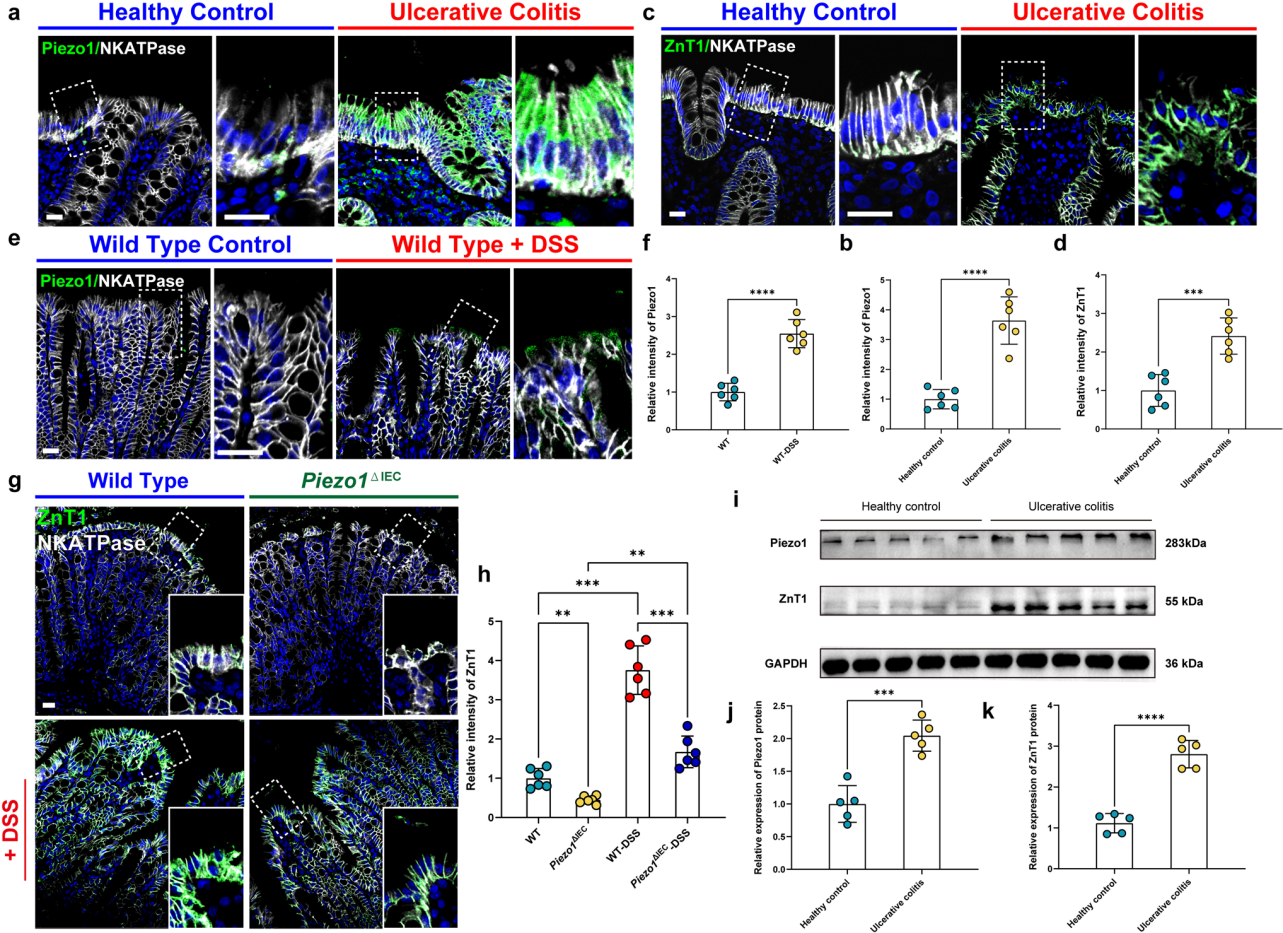
ZnT1 expression was elevated in colitis and correlated with Piezo1 expression. **a** Representative images showing Piezo1 IF staining of human colon tissues in healthy controls and UC patients. Scale bar: 20 μm. **b** Quantitative analysis of images shown in (a); *n* = 6 each group. **c** Representative images showing ZnT1 IF staining of human colon tissues in healthy controls and UC patients. Scale bar: 20 μm. **d** Quantitative analysis of images shown in (c); *n* = 6 each group. **e** Representative images showing Piezo1 IF staining of mouse colon tissues from groups WT and WT-DSS. Scale bars: 20 μm. **f** Quantitative analysis of images shown in (e); *n* = 6 each group. **g** Representative images showing ZnT1 IF staining of mouse colon tissues from groups WT, *Piezo1*^ΔIEC^, WT-DSS and *Piezo1*.^ΔIEC^-DSS. Scale bars: 20 μm. **h** Quantitative analysis of images shown in (g); *n* = 6 each group. **i** Representative images of WB showing Piezo1 and ZnT1 protein expression in human colon tissues in healthy controls and UCpatients; *n* = 5 each group. **j** and **k** Quantitative analysis of images shown in (i); *n* = 5 each group. Data are presented as mean ± SD (error bars). *p < 0.05, **p < 0.01, ***p < 0.001 and ****p < 0.0001

IF staining revealed significant elevation in both Piezo1 and ZnT1 protein expression in colonic mucosa samples from UC patients (Fig. 5a-d) and confirmed by WB analysis (Fig. 5i-k). Similarly increased expression of Piezo1 and ZnT1 protein was found in DSS-treated mice compared with controls shown by IF staining (Figs. 5e-h). In addition, IF staining showed a significant reduction in ZnT1 expression in *Piezo1*^ΔIEC^ mice compared to WT mice (Fig. 5g and h), suggesting the possibility that Piezo1 protein has an impact on ZnT1 expression.

### Piezo1 Regulated ZnT1 Expression and Zinc Homeostasis in Intestinal Epithelial Cells

**Fig. 6:**
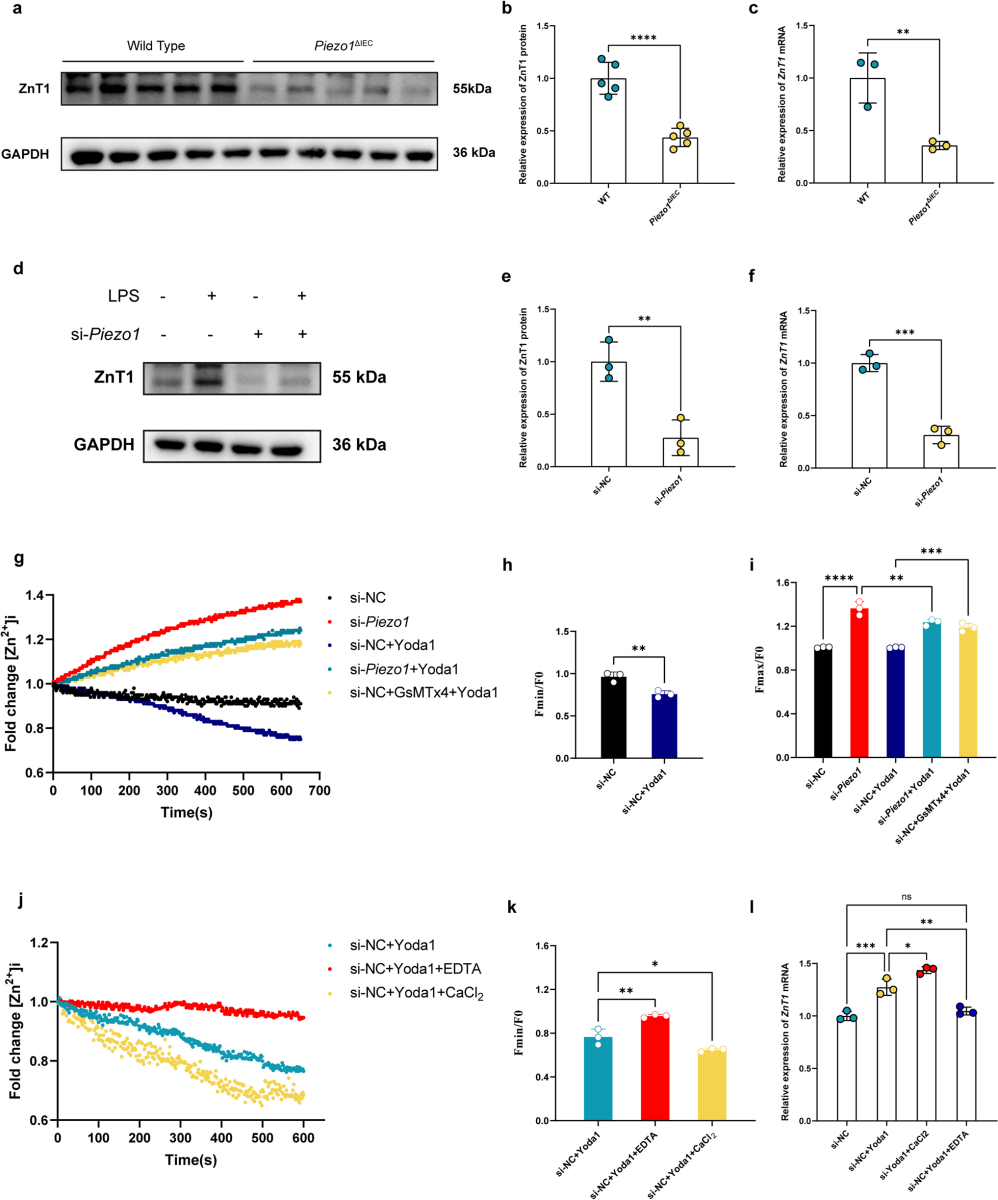
Piezo1 regulated ZnT1 expression and zinc homeostasis in intestinal epithelial cells. **a** Representative images of WB showing ZnT1 protein expression in mouse colon tissues from groups WT and *Piezo1*^ΔIEC^; *n* = 5 each group. **b** Quantitative analysis of images shown in (a); *n* = 5 each group. **c** qRT-PCR analysis of *ZnT1* mRNA expression in mouse colon tissues from groups WT and *Piezo1*^ΔIEC^; *n* = 3 each group. **d** Representative images of WB showing ZnT1 expression in Caco-2 cells transfected with si-*Piezo1* or si-NC and stimulated with LPS or not. **e** Quantitative analysis of ZnT1 protein expression in Caco-2 cells transfected with si-NC and si-*Piezo1* measured by WB; *n* = 3 each group. **f** qRT-PCR analysis of ZnT1 mRNA expression in Caco-2 cells transfected with si-NC and si-Piezo1; *n* = 3 each group. **g** Time-dependent changes in intracellular Zn^2+^ concentration ([Zn^2+^]i) quantified by fluorescence intensity in Caco-2 cells transfected with si-*Piezo1* or si-NC and treated with Yoda1 and GsMTx4; *n* = 12 each group. **h** Ratio of minimum intracellular zinc fluorescence intensity (F_min_) to value at start time (F_0_) in Caco-2 cells treated with Yoda1 or not. **i** Ratio of maximumintracellular zinc fluorescence intensity (F_max_) to value at start time (F_0_) in Caco-2 cells. **j** Time-dependent changes in [Zn.^2+^]_i_ quantified by fluorescence intensity in Yoda1 treated- Caco-2 cells treated with CaCl_2_ or EDTA or not; *n* = 12 each group. **k** Ratio of F_min_ to F_0_ in Yoda1 treated- Caco-2 cells treated with CaCl2 or EDTA or not. (l) qRT-PCR analysis of *ZnT1* mRNA expression in Yoda1-treated Caco-2 cells treated with CaCl_2_ or EDTA; *n* = 3 each group. Data are presented as mean ± SD (error bars). Data are presented as mean ± SD (error bars). *p < 0.05, **p < 0.01, ***p < 0.001 and ****p < 0.0001

In vitro experiments with the human colon cell-line, Caco-2, also indicated a positive correlation between Peizo1 and ZnT1 expression, consistent with the in vivo results from human and murine colitis samples. IEC-specific deletion of *Piezo1* in mice resulted in reduced expression of both ZnT1 mRNA and protein (Fig. 6a-c), and similar results was seen with *Piezo1* knocked down (si-*Piezo1*) Caco-2 cells (Fig. 6d-f). Piezo1 activation via agonist Yoda1 (si-NC + Yoda1) resulted in increasing Zn^2+^ efflux in Caco-2 cells, evidenced by decreasing intracellular Zn^2+^ levels compared with unstimulated (si-NC) cells shown by zinc imaging (Fig. 6g, i). Conversely, *Piezo1* knockdown (si-*Piezo1*) resulted in decreased Zn^2+^ efflux and intracellular Zn^2+^ accumulation (Fig. 6g, i). Treatment of si-*Piezo1* transfected Caco-2 cells with Yoda1 (si-*Piezo1* + Yoda1) produced a much milder response in terms of Zn^2+^ efflux (Fig. 6g, h), and similarly, inhibition of Piezo1 with inhibitor GsMTx4 abolished the Yoda1-induced intracellular Zn^2+^ efflux (si-NC + GsMTx4 + Yoda1 versus si-NC + Yoda1, Fig. 6g, i). These data indicate that Piezo1 had an impact on intracellular zinc homeostasis, and Zn^2+^ efflux was dependent on Piezo1 activity. Meanwhile, Ca^2+^ supplementation promoted Zn^2+^ efflux in Yoda1-induced Piezo1-activated Caco-2 cells (si-NC + Yoda1 + CaCl_2_ versus si-NC + Yoda1, Fig. 6j, k), while Ca^2+^ chelation inhibited Zn^2+^ efflux in Yoda1-treated Caco-2 cells (si-NC + Yoda1 + TPEN versus si-NC + Yoda1, Fig. 6j and k), suggesting that the enhanced Zn^2+^ efflux induced by Piezo1 activation is dependent on the elevation of intracellular Ca^2+^ concentration induced by enhanced Ca^2+^ influx. Ca^2+^ supplementation could also upregulate *ZnT1* mRNA expression in Yoda1-induced Piezo1-activated Caco-2 cells (si-NC + Yoda1 + CaCl2 versus si-NC + Yoda1, Fig. 6l), while Ca^2+^ chelation downregulated *ZnT1* mRNA expression (si-NC + Yoda1 + TPEN versus si-NC + Yoda1, Fig. 6l), indiating the Ca^2+^-mediated Piezo1 regulating ZnT1 could occur at the transcriptional level. Taken together, Piezo1 is suggested to be a positive regulator of the expression and function of the Zn^2+^ transporter, ZnT1, in IECs and modulates intracellular zinc homeostasis, and the regulatory effect of Piezo1 on Zn2 + efflux is Ca2 +—dependent.

## Discussion

UC is a long-term inflammatory disorder, characterized by frequent relapse, and incidence is on the increase. Therapeutic options are limited by incomplete understanding of the pathogenesis and further research is necessary to identify therapeutic and prognostic targets. A novel Piezo1-ZnT1-zinc axis which contributes to the maintenance of epithelial barrier integrity during colitis is presented in the current work.

Piezo1 suppression in IECs demonstrated protective effect against inflammation-induced epithelial barrier disruption both in vivo (DSS-treated *Piezo1*^ΔIEC^ mice) and in vitro (LPS-treated si-*Piezo1* Caco-2 cells), consistent with previous researches [4, 26]. Bioinformatic analysis indicated a correlation between Piezo1 expression and zinc homeostasis in the colonic mucosa of UC patients and consisted with transcriptomic analysis of *Piezo1*^ΔIEC^ mice (unpublished data). Top 50 DEGs between high- and low- Piezo1-expression group could be roughly divided into four categories: relating with zinc homeostasis, intestinal barrier function, inflammation and immune response, cell metabolism and proliferation. Zinc transporter genes (SLC30A10, SLC39A5) were markedly up-regulated in Piezo1 high-expression group, drawing our attention to zinc transport in IECs, especially the main Zn^2+^ efflux transporter ZnT1, though the dataset analyzed did not include this probe. Meanwhile, metallothionein (MT) family genes (MT1M, MT1G, MT1H, MT1E, MT1F, MT1X) responsible for intracellular zinc chelation also markedly up-regulated, which usually an adaptive response to cellular stress (e.g., inflammation, oxidative stress) sequestering cytosolic free Zn^2+^ [27]. Therefore, it can be indicated that promoted Zn^2+^ efflux (via ZnTs) coupled with enhanced intracellular zinc sequestration (via MTs) lead to disruption of cytosolic zinc homeostasis during inflammation in IECs with high Piezo1 expression. Specifically, pathways associated with Zn^2+^ transport across plasma membrane were enriched, led the focus of the work towards the plasma membrane Zn^2+^ efflux transporter, ZnT1 [9]. Zn^2+^ efflux and influx would normally be expected to be appropriately balanced to maintain intracellular homeostasis [9], it can be hypothesized that overexpression of Piezo1 associated with UC triggers excessive ZnT1-mediated Zn^2+^ efflux breaking this balance, producing a state of zinc dyshomeostasis in IECs. In accordance with this hypothesis, coordinated upregulation of Piezo1 and ZnT1 expression in colonic mucosa was observed in both human UC and murine colitis in our study. The positive regulatory impact of Piezo1 on ZnT1 expression was confirmed by using in vivo *Piezo1*^ΔIEC^ mouse model and the in vitro si-*Piezo1* Caco-2 cell model, in both of which Piezo1 suppression led to ZnT1 downregulation. Zinc imaging provided further evidence supporting Piezo1 altering intracellular zinc homeostasis, the activation of Piezo1 in Caco-2 cells by Yoda1 increased intracellular Zn^2+^ levels while the inhibition of Piezo1 by GsMTx4 or by si-*Piezo1* transfection reduced this effect. Therefore, we consider that these results support the view outlined above that the upregulation of Piezo1, known to be associated with UC, aggravated inflammatory damage of the intestinal epithelium via intracellular Zn^2+^depletion.

Piezo1 activation is known to trigger Ca^2+^ influx on a millisecond timescale following inflammatory stimulation and the sudden increase in intracellular Ca^2+^ disrupts the cation balance[4, 26, 28, 29], thus efflux of other intracellular cations including Zn^2+^ would be promoted to reverse the cation imbalance. Meanwhile, Piezo1 activation-induced Zn^2+^ efflux depends on intracellular Ca^2+^ elevation according to our study. Therefore a Ca^2+^-mediated regulatory axis can be proposed, the activation of Piezo1 triggers an increase in Ca^2+^ influx, the increased intracellular Ca^2+^ further mediates the subsequent elevation of Zn^2+^ efflux, thereby maintaining intracellular cation homeostasis in IECs, which might serve as an adaptive regulation strategy under inflammatory conditions. Our findings indicate that elevated intracellular Ca^2+^ serves as a critical downstream signal to promote Zn^2+^ efflux, likely through regulating the activity or expression of Zn^2+^ efflux transporters (e.g., ZnT1) in IECs. There are several potential mechanisms underlying the Ca^2+^-dependent regulatory mode of ZnT1: Piezo1 has been reported to activate efflux via potassium channels during inflammation [30] by a mechanism involving conformational change [31]. It is possible that a similar mechanism may allow the activation of ZnT1-mediated Zn^2+^ efflux in IECs, the increased intracellular Ca^2+^ triggered by Piezo1 activation may bind to intracellular domain of ZnT1, thus directly induces conformational change of ZnT1 protein and enhance its Zn^2+^ efflux activity, resulting in intracellular zinc deficiency which aggravates inflammation-induced barrier disruption. According to our findings, Ca^2+^ could alter *ZnT1* mRNA expression in Piezo1-activated IECs, indicating that Ca^2+^ signal may also act as a second messenger indirectly upregulates ZnT1 expression by activating transcription factors under inflammatory condition. Piezo1 activates Ca^2+^/calmodulin (CaM)/calmodulin-dependent protein kinase II (CaMKII) pathway triggering cAMP response element-binding protein (CREB) phosphorylation, the phosphorylated CREB forms a complex with CREB-binding protein (CBP) and binds with metal-response element-binding transcription factor (MTF)−1 [31–34], which can increase ZnT1 transcription via targeting metal-response element of its promoter [35]. Other responsive element in ZnT1 promoter may also play a role, Piezo1 can activate Ca^2^⁺/CaMKII/nuclear factor erythroid 2-related factor 2 (Nrf2) pathway under inflammation-induced oxidative stress [36, 37], ZnT1 transcription can also be enhanced by activation of Nrf2/antioxidant responsive element pathway [38].

The observations that Zn^2+^ chelation with TPEN exacerbated reductions of TEER and TJ proteins expression in LPS-treated Caco-2 cells and that Zn^2+^ supplementation with ZnCl_2_ produced an opposing effect support the view that intracellular zinc homeostasis is vital for maintenance of intestinal barrier integrity, in alignment with previous reports [12–14]. ZnT1 knockdown reproduced the alleviating effect of zinc supplementation on barrier disruption, highlighting the role of this transporter in the maintenance of zinc homeostasis in IECs. The activation of PI3K/AKT/mTOR signaling which stimulates TJ protein synthesis and epithelial regeneration [13] and inhibition of TLR4/NF-κB signaling to relieve barrier damage induced by inflammatory cytokine release [39] are both previously reported. Recent reports of noncanonical functions of ZO-1 and occludin in enhancing proliferation and survival of IECs [40] may also contribute to the alleviation of epithelial inflammatory damage. In addition, severe spontaneous enteritis due to stem cell death has been reported in IEC-specific *ZnT1* knockout mice [41], suggesting that a moderate level of ZnT1 expression is essential for maintaining gut homeostasis. Collectively, Piezo1-driven ZnT1 overexpression triggers excessive Zn^2+^ efflux, depleting cytoprotective zinc pools in IECs, thus aggravating inflammation-induced epithelial barrier disruption. Moreover, our findings suggested that suppression of the Piezo1-ZnT1-zinc axis can be expected to alleviate intestinal damage during UC.

The therapeutic role of zinc supplementation in IBD management has been widely recognized [11, 42] however clinical usage has been limited by the lack of clinical evidence and standardized guidelines [43, 44]. Traditional oral zinc supplements, such as zinc gluconate and zinc sulfate, have been reported to have low bioavailability [45]. Although there is hope for emerging nano-zinc supplements, none has yet been prescribed to patients [46]. It is also the case that zinc supplementation alone is unlikely to rectify intracellular zinc dyshomeostasis in the presence of continuing excessive Zn^2+^ efflux due to pathological Piezo1-ZnT1 signaling, thus a synergistic approach of Piezo1/ZnT1 inhibition plus zinc delivery may overcome these problems. So far, none of the existing Piezo1 and ZnT1 inhibitor has yet been clinically approved. Ginsenoside Rb1 and GsMTx4 are Piezo1 inhibitors that have been used in animal models, and GsMTx4 is reported to ameliorated DSS-induced colitis [47, 48]. However, main obstacles faced in clinical translation include insufficient binding affinity due to the ligand-binding structure not fully decoded, and systemic toxicity due to broad distribution and functions [48]. No specific ZnT1 inhibitor has yet been discovered and the design of drugs to target ZnT1 is limited by the same difficulties as for Piezo1. Notwithstanding remaining obstacles, targeting the Piezo1-ZnT1-zinc axis is a promising therapeutic strategy for UC deserves further exploration.

Our study establishes Piezo1 as a crucial regulatory factor for zinc homeostasis in IECs by positively modulating ZnT1-mediated Zn^2+^ efflux, indicating a direct linkage of mechanical stresses with the homeostasis of vital metal ion. Normally cells coordinate ion fluxes (e.g., Ca^2+^ influx and Zn^2+^ efflux) to maintain intracellular ion homeostasis, however it can be pathologically amplified under sustained stimulation such as UC. Piezo1 activation by inflammation-induced mechanical stimuli triggers rapid Ca^2+^ influx [3, 5, 26], in turn upregulates expression and activity of ZnT1 via Ca^2+^-mediated conformational change and transcriptional reprogramming, leading to excessive Zn^2+^ efflux, intracellular Zn^2+^ depletion, and ultimately compromised barrier integrity.

While this work presents initial insights into Piezo1 regulating zinc homeostasis in UC, several limitations warrant acknowledgment. Firstly, DSS-induced murine colitis and LPS-stimulated Caco-2 cells are simplified models for investigating IEC-specific acute inflammatory responses, which may not.

adequately recapitulate the complexity of UC pathophysiology. Future use of chronic colitis models, such as IL-10 knockout or T-cell transfer mice, and human organoids may give a better simulation of chronic immune dysregulation of the barrier microenvironment in UC patients [49–51]. Secondly, our study focused on short-term responses in IECs following Piezo1-ZnT1 modulation and inflammatory stimuli, while tracking of ZnT1 expression, dynamics of zinc homeostasis and zinc-dependent processes would be required for longer-term assessments. Thirdly, whether Piezo1-driven ZnT1 modulation is conducted by direct conformational coupling [31] or transcriptional reprogramming such as Ca^2+^-dependent MTF-1/Nrf2 targeting ZnT1 promoter [35, 36, 38, 52] remains unclear, further researches involving ChIP sequencing and Co-IP assays are needed to reveal the precise molecular mechanism. Fourthly, intracellular Zn^2+^ is not uniformly distributed but localized to subcellular pools with distinct functions [53], however in this study we only measured total intracellular Zn^2+^ concentration but did not map Piezo1-ZnT1’s impact on subcellular zinc pools, thus it remains unclear whether Piezo1-driven Zn^2+^ efflux depletes specific pools (e.g., mitochondrial zinc) that are critical for IEC survival and barrier function. Organelle specific zinc fluorescent probe and high-resolution nanoscale secondary ion mass spectrometry might be helpful. Besides, assessment of the contribution of ZIP family transporters in compensating for Zn^2+^ depletion by enhancing Zn^2+^ influx was outside the scope of the present work, which remains to be investigated. Lastly, the small sample size of patient specimens precluded stratified analysis by clinical characteristics of disease severity, stage or location, multicenter cohorts enable establishment of correlation between Piezo1-ZnT1 dysregulation and these features.

## Conclusion

Our study reveals that Piezo1-driven ZnT1 upregulation disrupted zinc homeostasis by enhancing Zn^2+^ efflux in IECs, resulting in cytosolic zinc depletion and compromised epithelial barrier integrity. Suppression of the Piezo1-ZnT1-zinc axis alleviated inflammation-induced barrier disruption, suggesting a promising therapeutic target for UC.

## Supplementary Information

Below is the link to the electronic supplementary material.

Supplementary file1 (DOCX 13561 KB)

## Data Availability

All data supporting the conclusions of this article are included within the article and supplementary materials. The data are available from the corresponding author upon reasonable request.
