# Supplementary material for: Piezo1 Regulates ZnT1-Mediated Zinc Homeostasis in Ulcerative Colitis
Source: Inflammation. 2026 Jan 26;49(1):68. doi: 10.1007/s10753-025-02448-5 (PMC12891053; doi:10.1007/s10753-025-02448-5)
Supplement: Supplementary file 1 — Supplementary file1 (DOCX 13561 KB) [file 10753_2025_2448_MOESM1_ESM.docx]

**Supplementary Tables**

**Table S1. Baseline characteristics of UC patients and healthy Controls​**

| **Characteristic** | **UC patients**  **(n=6)** | **Healthy controls**  **(n=6)** |
| --- | --- | --- |
| **Age (years), mean ± SD** | 44.33±6.13 | 42.83±8.59 |
| **Male, n** | 3 | 3 |
| **Extend of disease** ^a^ |  |  |
| Proctitis, n | 0 | **-** |
| Left-sided colitis, n | 5 | **-** |
| Extensive colitis, n | 1 | **-** |
| **Mayo score, mean ± SD** | 7.67±1.97 | **-** |
| **Severity** ^b^ |  |  |
| Remission, n | 0 | **-** |
| Mild, n | 1 | **-** |
| Moderate, n | 4 | **-** |
| Severe, n | 1 | **-** |
| **Treatment** |  |  |
| Aminosalicylates | 0 | **-** |
| Immunosuppressor | 0 | **-** |
| None | 6 | **-** |

1. According to Montreal classification. b. Based on Mayo score, remission (< 2), mild (3-5), moderate (6-10), severe (> 11).

**Table S2. DAI scoring criteria**

| **Parameter** | **Score** | **Definition** |
| --- | --- | --- |
| **Weight loss** | 0 | < 1% loss |
|  | 1 | 1%-5% loss |
|  | 2 | 5%-10% loss |
|  | 3 | 10%-15% loss |
|  | 4 | > 15% loss |
| **Stool consistency** | 0 | Well-formed pellets |
|  | 1 | Slightly loose stools |
|  | 2 | Loose stools |
|  | 3 | Watery diarrhea |
|  | 4 | Severe diarrhea with mucus |
| **Fecal bleeding** | 0 | No blood (negative fecal occult blood test) |
|  | 1 | Mild bleeding (trace blood in stool) |
|  | 2 | Moderate bleeding (visible blood streaks) |
|  | 3 | Gross bleeding (blood-coated stool) |
|  | 4 | Severe hemorrhage |

DAI = (weight loss + stool consistency + fecal bleeding) /3

**Table S3. Oligonucleotide sequences of si-RNA**

| **Target RNA** | **Sequence**(5’-3’) |
| --- | --- |
| **Piezo1** | Sense: AAGAAAGUUCAUGCGCUGCCC-dTdT |
|  | Antisence: GGCAGCGCAUGAACUUUCUUU-dTdT |
| **ZnT1-001** | Sense: AGUGUAGGCUCUAAAUCAA-dTdT |
|  | Antisence: UUGAUUUAGAGCCUACACU-dTdT |
| **ZnT1-002** | Sense: CAACCUAUCCAUUACUUAA-dTdT |
|  | Antisence: UUAAGUAAUGGAUAGGUUG-dTdT |
| **ZnT1-003** | Sense: AGAACUUCGAAAUGUUGAA-dTdT |
|  | Antisence: UUCAACAUUUCGAAGUUCU-dTdT |

**Table S4. Inflammation and structural damage scoring criteria**

| **Parameter** | **Score** | **Definition** |
| --- | --- | --- |
| **Inflammation severity** | 0 | No or occasional inflammatory cells (in lamina propria) |
|  | 1 | Increased inflammatory cells in lamina propria |
|  | 2 | Inflammatory cell infiltration extending to submucosa |
|  | 3 | Transmural inflammatory infiltration |
| **Inflammation extent** | 0 | No inflammation |
|  | 1 | Focal involvement (< 25% of intestinal segment) |
|  | 2 | Regional involvement (25-50%) |
|  | 3 | Extensive involvement (> 50%) |
| **Crypt damage** | 0 | Intact crypts |
|  | 1 | Basal 1/3 damage |
|  | 2 | Basal 2/3 damage |
|  | 3 | Only surface epithelium intact |
|  | 4 | Complete crypt loss |
| **Percentage involvement** | 0 | < 1% |
|  | 1 | 1%-25% |
|  | 2 | 26%-50% |
|  | 3 | 51%-75% |
|  | 4 | > 75% |

Histological score = inflammation severity + inflammation extent + crypt damage + percentage involvement

**Table S5. Oligonucleotide sequences for qRT-PCR**

| **Primer** | **Sequence** (5’-3’) |
| --- | --- |
| **Piezo1** | Forward: CGTCTTCGTGGAGCAGATG |
|  | Reverse: GCCCTTGACGGTGCATAC |
| **ZnT1** | Forward: CTCGCGTTAAGAGCACCCG |
|  | Reverse: CAATTTCAGCCCGTTGGAGTT |
| **GAPDH** | Forward: GACAGTCAGCCGCATCTTCT |
|  | Reverse: GCGCCCAATACGACCAAATC |
| **ZO-1** | Forward: TCACCTTGACCACAGCAAAG |
|  | Reverse: TGCTGGTCTCTCAATCTGCT |
| **Occludin** | Forward: GGCGAAGACACCATCTCCA |
|  | Reverse: TGGGTAAAAGGCAGCAAAGC |

**Table S6. Top 50 differentially expressed genes (DEGs) between low and high Piezo1 expressing UC patients**

| **Up-regulated DEGs** | **Down-regulated DEGs** |
| --- | --- |
| MS4A12; TMIGD1; HMGCS2; CLCA1; CYP2B6; B4GALNT2; CA1; ZG16; MIR15A; PCK1; HEPACAM2; ABCG2; MIR215; CWH43; AQP8; MT1M; CYP2B7P; MIR222; TMEM236; SLC26A2; RSAD2; BEST2; PADI2; ANPEP; SLC30A10; GUCA2A; MEP1B; OTOP2; LRRC19; DHRS11; CHP2; ABCB1; ADH1C; ARL14; ITLN1; MIR221; ENTPD5; TRPM6; CLCA4; ACSF2; MIR194-1; SLC9A2; SULT1A2; CLDN8; CD177; HPGD; SLC51B; MEP1A; FER1L6; SLC17A4 | CHI3L1; TCN1;OR2J3; LCN2; MMP7; ROCK1P1; DUOX2; RGS5; OR4K2; DUOXA2; MTND2P28; SLCO1B3; VWF; NMUR2; CXCL1; IGH; F2RL2; LHFPL6; OR8G1; OR2L8; IRF4; IGKV2-24; ADGRL4; RGS1; PECAM1; TIMP1;KYNU; MGP; C4A; POSTN; IGKC; KDR; COL6A3; IGLC1; MZB1; IGFBP5; SPARCL1; MXRA5; CD79A; CD34; OSMR; REXO1L1P; EDNRA; LAX1; PCDH18; COL1A2; HLA-DQB1; FLT1; CD180; PTCH2 |

**Table S7. Highlighted pathway in KEGG enrichment analysis**

| **ID** | **Description** | **Enriched genes** |
| --- | --- | --- |
| hsa04978 | Mineral absorption | ATP1B3/ VDR/ MT1M/ MT1G/ MT1H/ MT1E/ MT1F/ CLCN2/ MT1X/ TRPM6/ HEPH/ SLC9A3/ SLC26A3 |

**Table S8. Highlighted pathways in GO enrichment analysis**

| **ID** | **Description** | **Enriched genes** |
| --- | --- | --- |
| GO:0071294 | cellular response to zinc ion | P2RX4/ MT1M/ MT1G/ MT1H/ MT1E/ MT1F /MT1X /GLRA2 |
| GO:0006882 | intracellular zinc ion homeostasis | MT1M/ MT1G/ MT1H/ MT1E/ SLC30A10/ MT1F/ MT1X |
| GO:0071578 | zinc ion transport across plasma membrane | SLC39A11/ SLC39A5 |

**Supplementary Figures**

**
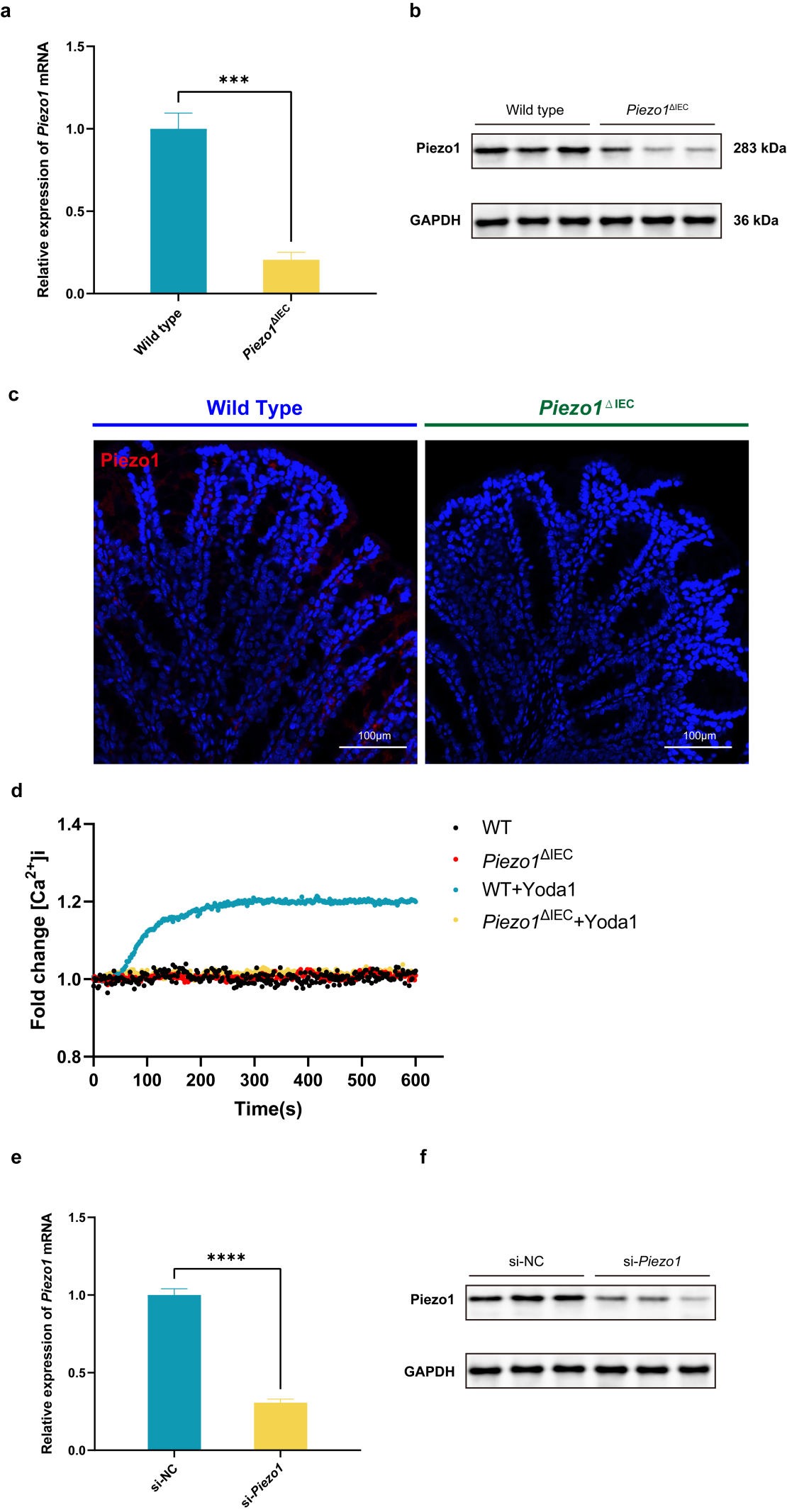
**

**Fig S1. Validation of Piezo1 suppression *in vitro* and *in vivo*.** (a-c) Piezo1 expression in wild type and *Piezo1*^ΔIEC^ mice colon tissues detected by qRT-PCR (a), WB (b), IF staining (c). (d) Time-dependent changes in intracellular Ca^2+^ concentration ( [Ca^2+^]i ) quantified by fluorescence intensity in Yoda1-treated IECs from WT or *Piezo1*^ΔIEC^ mice; n = 12 each group. (e,f) Piezo1 expression in si-NC and si-*Piezo1* transfected Caco-2 cells detected by qRT-PCR (e) and WB (f).

**
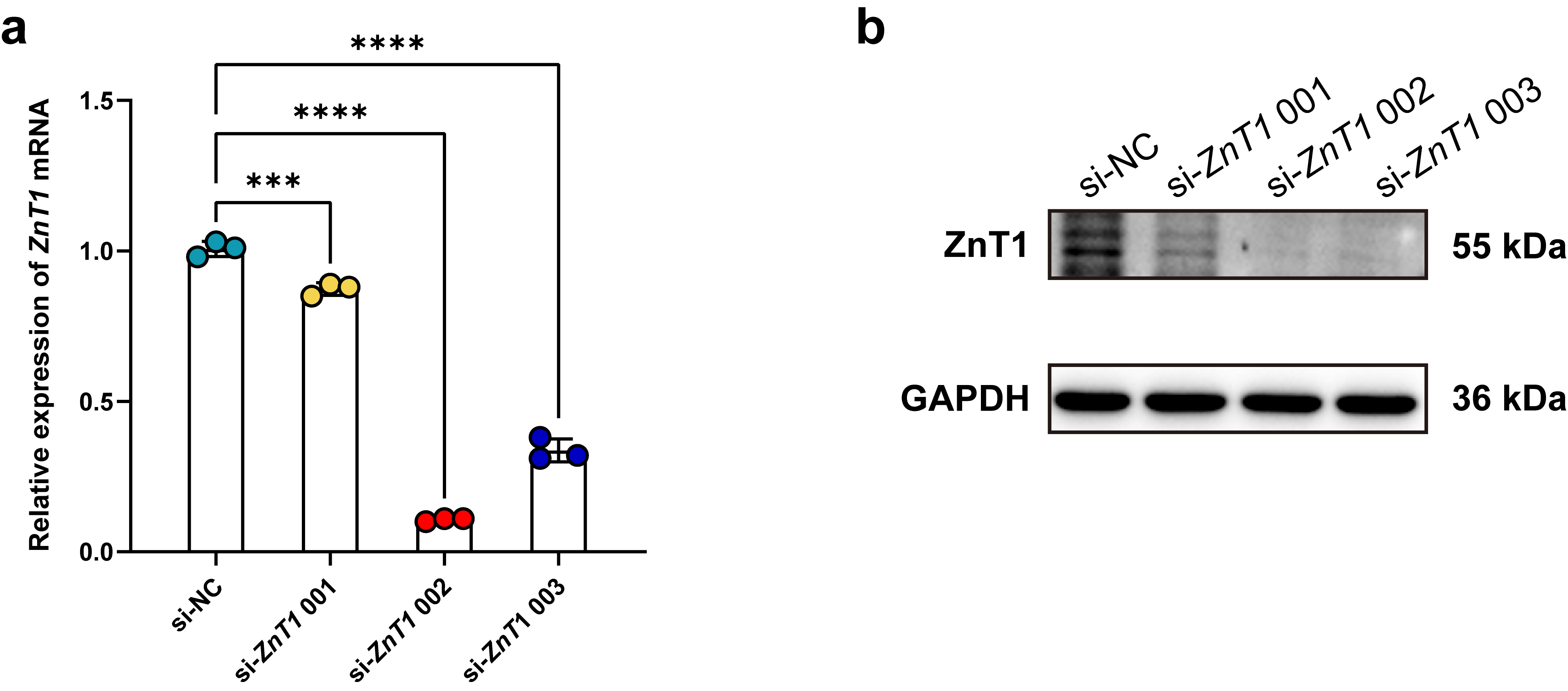
**

**Fig S2. Validation of ZnT1 knockdown in Caco-2 cells.** (a) qRT-PCR analysis of *ZnT1* mRNA expression in Caco-2 cells transfected with three different si-RNAs targeting ZnT1: si-*ZnT1* 001, si-*ZnT1* 002 and si-*ZnT1* 003. (b) Representative images of WB of ZnT1 protein expression in Caco-2 cells transfected with three different si-RNAs targeting ZnT1: si-*ZnT1* 001, si-*ZnT1* 002 and si-*ZnT1* 003.

**
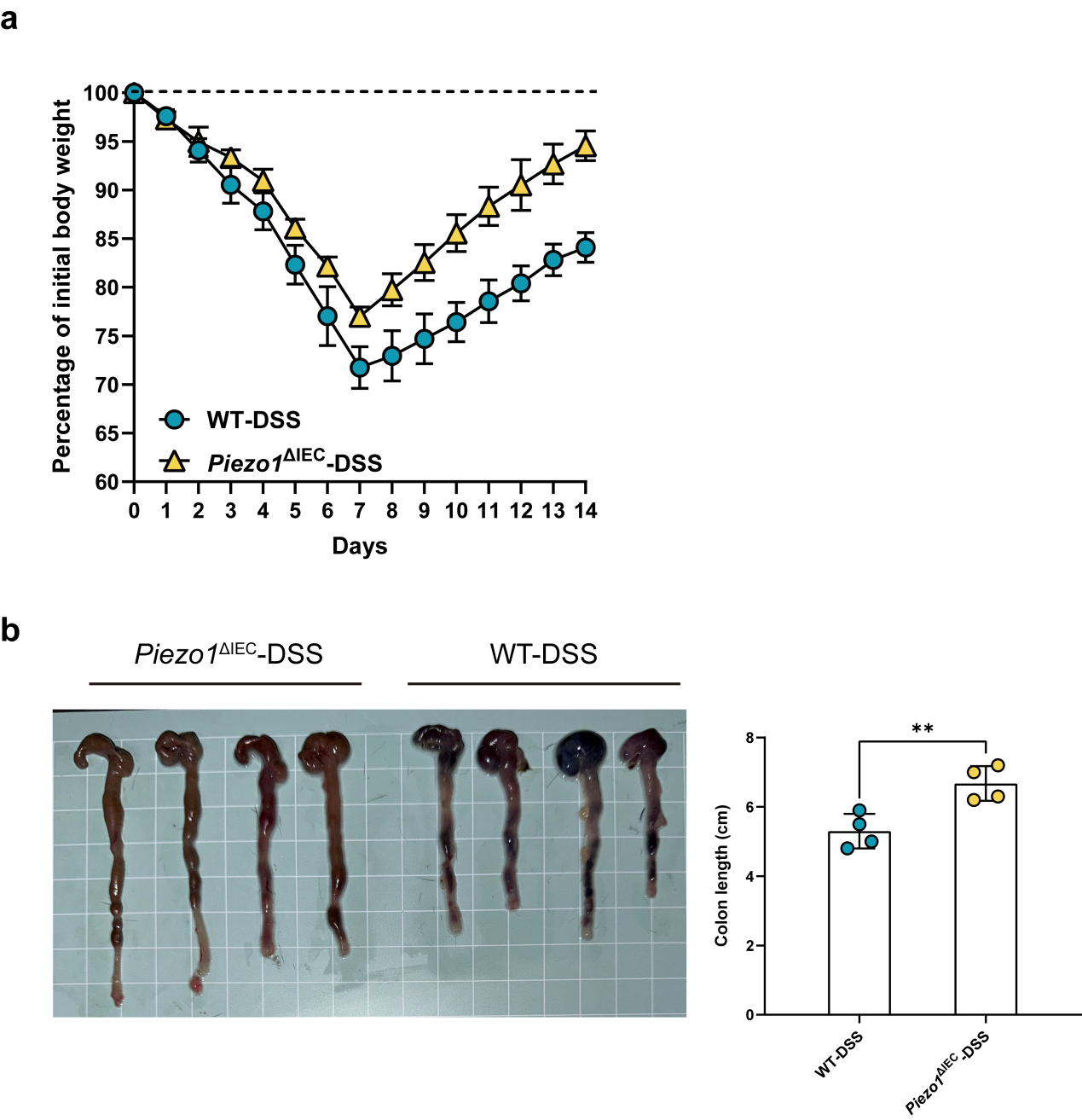
**

**Fig S3. Body weight changes and colon length of WT and Piezo1^ΔIEC^ mice treated with DSS for 7 days followed by 7 days of recovery.** (a) Time course of body weight changes of mice from groups WT-DSS and *Piezo1*^ΔIEC^-DSS over 7 days, the DSS administration was withdrawn at day 8; n=4 each group. (b) Representative images and colon length statistics of mice from groups WT-DSS and *Piezo1*^ΔIEC^-DSS sacrificed at day 14; n=4 each group.
